# Suitability of testers to characterize provitamin a content and agronomic performance of tropical maize inbred lines

**DOI:** 10.3389/fgene.2022.955420

**Published:** 2022-08-08

**Authors:** Abdoul-Raouf Sayadi Maazou, Victor O. Adetimirin, Melaku Gedil, Silvestro Meseka, Wende Mengesha, Abebe Menkir

**Affiliations:** ^1^ Pan African University Life and Earth Sciences Institute (including Health and Agriculture), University of Ibadan, Ibadan, Nigeria; ^2^ International Institute of Tropical Agriculture (IITA), Ibadan, Nigeria; ^3^ Department of Crop and Horticultural Sciences, University of Ibadan, Ibadan, Nigeria

**Keywords:** testers, testcrosses, inbreds, provitamin A, carotenoids, tropical maize

## Abstract

Vitamin A deficiency poses health risks for children, pregnant women, and nursing mothers in sub-Saharan Africa (SSA) and Southeast Asia. Provitamin A–biofortified maize varieties can contribute to minimizing the adverse effects of vitamin A deficiency in areas where maize is a staple food crop. Identifying suitable testers is important to breed provitamin A–biofortified hybrid maize. This study was therefore conducted to 1) assess the suitability of maize inbred lines with contrasting levels of provitamin A (one with high and one with low provitamin A concentration) to assess the combining ability of maize inbred lines in accumulating provitamin A and other carotenoids, and grain yield, 2) confirm the mode of inheritance of provitamin A and grain yield, and 3) identify promising inbred lines with desirable combining ability effects for use to develop high-yielding provitamin A–biofortified hybrids. The inbreds crossed to the two inbred testers were evaluated in four environments for the carotenoid content and eight environments for the agronomic performance. The combined analysis of variance revealed a significant genetic variation among the testcrosses for all carotenoids, grain yield, and other agronomic traits. The mode of inheritance for grain yield, other agronomic traits, provitamin A, and other carotenoids was regulated by both additive and non-additive gene effects with a prominence of additive gene effects. The high provitamin A tester that displayed positive GCA effects for β-carotene and provitamin A content, broader agronomic performance of testcrosses, and higher levels of provitamin A in testcrosses can be considered suitable for breeding programs developing provitamin A–biofortified hybrids. The inbred lines TZI2012, TZI2142, TZI2130, TZI2065-2, TZI2161, TZI2025, TZI1278, TZI1314, TZI1304, and TZI2032 with positive GCA effects for grain yield and provitamin A content could be used as parental lines to develop source population of new inbred lines and high-yielding hybrids with elevated levels of provitamin A. The best performing hybrids are promising for release as high-yielding provitamin A maize hybrids after further evaluations.

## Introduction

Vitamin A deficiency (VAD) is one of the highest health risks, with the largest prevalence in sub-Saharan Africa (SSA) and Southeast Asia (WHO, 2009). The prevalence of VAD in SSA has been estimated to be 48% in 2013 (Stevens et al., 2015). Vitamin A is an essential micronutrient needed by the human body to improve vision and immunity from infectious diseases such as malaria, diarrhea, and measles (Rice et al., 2004). As humans are not able to synthesize vitamin A in their bodies, they need to obtain it from plants and other sources in their diets.

White maize (*Zea mays* L.) is consumed as a staple food in many countries in SSA such as South Africa, Nigeria, Ethiopia, Tanzania, Kenya, Malawi, Mali, and Zambia, where VAD is most prevalent. Although over 90% of the maize produced in Africa is white (Khumalo et al., 2011), the production of orange maize is increasing due to rising promotion for its intake due to higher carotenoid content (Ekpa et al., 2019). In addition, an acceptability study conducted by Muzhingi et al. (2008) revealed that consumers like the flavor of provitamin A (PVA) maize, and they do not object to its orange color. The development of maize varieties with enhanced levels of provitamin A carotenoids (PVA) has, thus, been considered an important and affordable approach to mitigate the negative impact of VAD in SSA. Biofortification of maize with provitamin A has been achieved because of the presence of considerable genetic variation in PVA concentrations (Ortiz-Monasterio et al., 2007; Menkir et al., 2008; Pixley et al., 2013).

In many African countries, hybrid maize varieties have been grown for decades because of their high yield potential and other desirable agronomic features such as uniformity of hybrid plants, large and uniform cobs, and resistance to pests and diseases. In SSA, 17% improvement in maize yield due to the adoption of hybrid varieties was reported by Suri (2011). Advances in understanding the breeding value of suitable testers are, therefore, important for accurate assessment of the combining ability of new provitamin A–enriched maize inbred lines to select promising parents for developing productive hybrids and source populations for more robust inbred lines (Hallauer and Lopez-Perez, 1979). Commonly used testers can be classified into two groups: broad genetic base testers and narrow genetic base testers. Broad genetic base testers such as open-pollinated cultivars and synthetic cultivars can be used to assess general combining ability (GCA) effects of lines under evaluation, whereas narrow genetic base testers can be used to assess both GCA and specific combining ability (SCA) effects (Acquaah, 2012). Testers can also be classified based on their frequency of favorable alleles of a trait of interest. Several studies have been conducted and provided different recommendations about selecting desirable testers for maize breeding programs. Some recommend selecting testers with a low frequency of favorable alleles to identify lines with a high frequency of favorable alleles for developing productive hybrids (Hallauer and Miranda Filho, 1995; Hallauer et al., 2010) whereas others propose the use of inbred testers with high frequencies of favorable alleles to select parents of hybrids with superior agronomic performance for direct commercialization (Abel and Pollak, 1991; Hallauer and Carena, 2009). To the best of our knowledge, however, suitable testers that can be used for assessing the combining ability of provitamin A–enriched maize inbred lines to develop superior hybrids have not been reported. Such information can help in identifying new parental lines in a breeding program to generate hybrids combining high yield potential with elevated provitamin A content.

Lines by tester crosses have been extensively used not only to identify suitable testers but also to determine the mode of inheritance of traits, including carotenoids. Some studies found additive gene action is more important in regulating the PVA carotenoid content in maize (Menkir et al., 2014; Owens et al., 2014; Suwarno et al., 2014), while another study reported the preponderance of non-additive gene action in controlling PVA concentrations (Halilu et al., 2016). These inconsistent findings highlight the need to further assess the type of gene action controlling PVA concentration in maize. In addition, identifying parents with desirable combining ability effects for both provitamin A content and agronomic traits may facilitate simultaneous increases in grain yield and provitamin A levels in new parental lines and their hybrids.

The maize breeding program at the International Institute of Tropical Agriculture (IITA) developed many provitamin A–enriched maize inbred lines by introgressing favorable alleles of high β-carotene content from 12 exotic lines into elite tropical inbred lines (Menkir et al., 2015). The provitamin A–enriched maize lines have been further crossed to tropical introduced maize inbred lines of diverse origin to broaden the genetic base of the existing germplasm and develop new parents with greater concentrations of provitamin A and other carotenoids. When such new inbred lines are developed from crosses between elite lines and introduced lines with unknown heterotic affinities, crossing the new lines with known testers and evaluating the resulting hybrids in multiple locations can determine their usefulness as parents to develop source populations and high-yielding hybrids with greater levels of provitamin A.

The present study was, therefore, conducted to 1) assess the suitability of maize inbred lines with contrasting levels of provitamin A for assessing the combining ability of maize inbred lines in accumulating provitamin A and other carotenoids, and grain yield, 2) confirm the mode of inheritance of provitamin A and grain yield, and 3) identify promising inbred lines with desirable combining ability effects for use to develop high-yielding hybrids with much higher levels of provitamin A.

## Materials and methods

### Plant material and experimental design

A total of 60 PVA maize inbred lines developed at the Maize Improvement Program of IITA and two inbred testers with different levels of PVA concentration were used in this study (Supplementary Table S1). The PVA lines were selected based on variation in provitamin A content (5.4–51.7 μg/g) and the presence of different temperate donor parents of high β-carotene as well as tropical recipients (Maazou et al., 2021). The two testers represent two complementary heterotic groups of the IITA maize breeding program. Tester 1 represents heterotic group A while tester 2 represents heterotic group B (Menkir et al., 2004; Zebire et al., 2021; Maazou et al., 2022). The 60 lines were crossed to the two testers using a line × tester mating design to produce 120 testcrosses during two dry seasons (December 2019 to April 2020 and December 2020 to April 2021) at IITA’s research field in Ibadan (7°29′11.99″N, 3°54′2.88″E, altitude 190 masl), Nigeria. The hybrid seeds from reciprocal crosses were bulked to obtain a sufficient quantity of seeds for multi-environment evaluations, and reduce the cost of carotenoid analysis. The decision to bulk reciprocal crosses was made considering the fact that reciprocal effects are not important for provitamin A content in maize grain (Ortiz-Covarrubias et al., 2019). The 120 testcrosses and a hybrid from a cross between the two testers as well as three commercial hybrids, namely, Ife Hybrid-3, Ife Hybrid-4, and Oba Super 2 included as checks were evaluated at four locations in Nigeria, Ikenne (3°42′ E, 6°54′ N, 30 masl), Saminaka (8°39′ E, 10°34′ N, 760 masl), Zaria (7°45′ E, 11°8′ N, 622 masl), and Mokwa (5°4′ E, 9°18′ N, 457 masl) in 2020 and 2021 during the main cropping seasons (June to November), making a total of eight environments. Agronomic data were collected from the eight environments, while carotenoids were evaluated in four environments (Ikenne and Saminaka in 2020 and 2021).

The trial was arranged in a 31 × 4 alpha-lattice design with two replications. Experimental plots were single 5 m long rows spaced 0.75 m apart with a plant-to-plant spacing of 0.25 m within a row, giving a population density of 53,000 plants ha-1. The fertilizer NPK 15:15:15 was applied at the rate of 60 kg N ha-1, 60 kg P ha-1, and 60 kg K ha-1 during planting; urea (46-0-0) was used to apply 60 kg N ha-1 4 weeks after planting. Herbicides (Primextra and Gramazone) were also applied 2 days after planting as recommended for optimum maize production.

### Agronomic data collection

Plant height (PHT), ear height (EHT), days to anthesis (DYANTH), days to silking (DYSK), ear aspect (EASP), plant aspect (PASP), grain weight, and grain moisture were recorded from each plot. The data collection was carried out following the method described by Menkir et al. (2014). Briefly, PHT and EHT were measured in cm as the distance from the base of the plant to the first tassel branch and the node bearing the upper ear, respectively. DYANTH and DYSK were recorded as number of days from planting to the date when 50% of the plants in a plot had tassels shedding pollen and emerged silks, respectively. Anthesis–silking interval (ASI) was calculated as the difference between DYSK and DYANTH. Ear aspects were scored on a 1 to 5 scale, where 1 represented clean, well-filled, uniform and larger ears, while 5 represented diseased, poorly filled, variable, and smaller ears. Plant aspect was also scored on a 1 to 5 scale, where 1 represented uniform, clean, vigorous, and good overall phenotypic appeal, while 5 represented weak, diseased, and poor overall phenotypic appeal. Harvested ears were shelled, and the grain moisture content of shelled grains was measured using a portable DICKEY-john moisture tester. The grain weight and moisture content were used to compute grain yield adjusted to 15% moisture.

### Carotenoid analysis

In each year of field evaluation, composite grain samples for carotenoid analysis were taken from harvested self-pollinated ears of five representative plants in each plot at two locations (Ikenne and Saminaka). Carotenoids were extracted from the maize kernels and quantified by high-performance liquid chromatography (HPLC, Water Corporation, Milford, MA, United States) at the Food and Nutrition Laboratory at IITA. The extraction protocol and carotenoid analysis used were based on the method described by Howe and Tanumihardjo (2006). Briefly, 0.6 g finely ground sample of each entry in two replicates was transferred into a 50 ml glass centrifuge tube to which 6 ml of ethanol and 0.1% butylated hydroxyl toluene were added, vortexed for 15 s, and incubated at 85°C in a water bath for 5 min. After that, 500 μl of 80% potassium hydroxide (w/v) was added to each sample, vortexed for 15 s, and incubated at 85°C in a water bath for 10 min with vortexing at about 5 min intervals. The samples were then immediately placed on ice and 3 ml ice-cold deionized water was added to each of them, vortexed for 15 s, and 200 μl internal standard β-Apo- 8′-carotenal and 4 ml hexane were added. After vortexing and centrifugation, the top hexane layer formed was transferred into a new test tube. The hexane extraction was repeated thrice, adding 3 ml hexane each time. The samples were allowed to dry down completely under nitrogen gas using a concentrator (Organomation Associates, Inc., Berlin, MA, United States) and reconstituted in 1 mL of 50:50 methanol:dichloroethane and vortexed for 10 s. For each sample, 50 μl aliquots of each extract were injected into the HPLC system and run for major carotenoids based on the calibration of the standard of each carotenoid. Carotenoids were separated by a C30 column (4.6 × 250 mm; 3 μm) eluted by a mobile phase using methanol/water (92: 8 v/v) as solvent A and 100% methyl tertiary-butyl ether (MTBE) as solvent B. The flow rate of solvent was 1 mL/min, and absorbance was measured at 450 nm for carotenoid detection. Chromatograms were extracted after the runs and major carotenoids were identified.

Total carotenoid was calculated as the sum of concentrations of α-carotene, lutein, β-carotene, β-cryptoxanthin, and zeaxanthin. PVA was calculated as the sum of β-carotene and half of each of β-cryptoxanthin and α-carotene content (US Institute of Medecine, 2001). All concentrations were described in μg g-1 dry weight (DW).

### Data analysis

The combined analysis of variance (ANOVA) was performed following the line × tester procedure of Singh and Chaudhary (1977) using the Proc mixed procedure in SAS version 9.4 (SAS Institute Inc, 2012). In the combined analysis, each location-year combination was considered an environment. Hybrids were considered as fixed effects, while environment, replication (environment), block (replication × environment), and environment × hybrid were considered as random effects in the linear model. The hybrid mean square was further partitioned into lines, testers, line × tester, environment × line, environment × tester, and environment × line × tester effects using a line × tester analysis. The genetic variance estimates resulting from combined analysis of variance of testcross means of each tester obtained from the eight environments were used to assess the usefulness of the testers (Afolabi et al., 2021). Phenotypic and genotypic correlation coefficients between agronomic traits and carotenoids were estimated using META-R v6.03 (Alvarado et al., 2020), developed at CIMMYT, Mexico.

Standard heterosis (H) was also calculated for each testcross using the formula of Fan et al. (2016):

$$H=100\%\times \left(F_{1}-CK\right)/CK,$$

where F1 is the grain yield of a testcross and CK is the grain yield of the hybrid between the two testers (T1 × T2).

After exclusion of the checks, the general combining ability (GCA) and specific combining ability (SCA) effects of the parental inbred lines and the variance components for each trait were calculated with analysis of genetic design (AGD-R, V.5.0) (Rodríguez et al., 2018). The restricted maximum likelihood method (REML) was used to estimate the variance components (Rodríguez et al., 2018). The relative importance of GCA and SCA effects was estimated using the formula of Baker (1978) as follows:

$$Baker\;ratio=\frac{2MS_{GCA}\;}{2MS_{GCA}+2MS_{SCA}},$$

where MSGCA and MSSCA are the mean squares for GCA and SCA, respectively. The closer the ratio to unity, the greater the predictability of hybrid performance based on GCA effects alone (Baker, 1978).

## Results

### Variations in carotenoid content and agronomic traits among testcrosses

**FIGURE 1:**
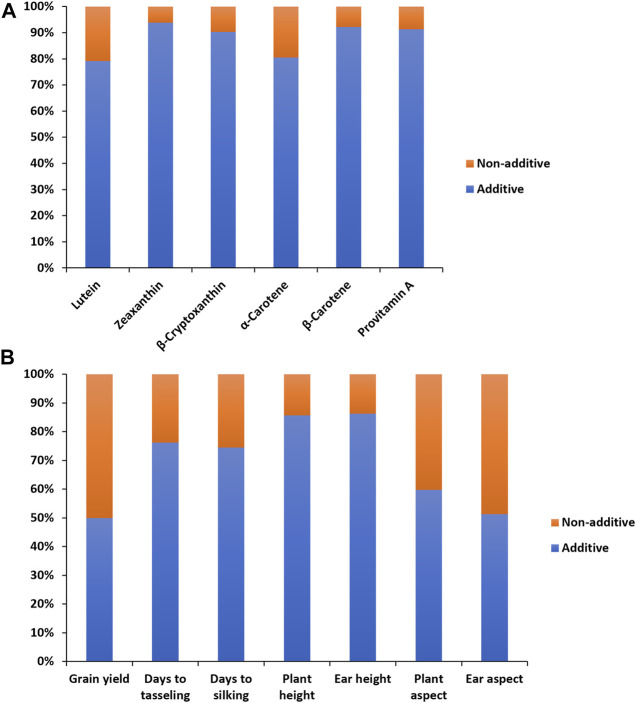
Proportion of additive (lower bar) and non-additive (upper bar) genetic variances for provitamin A and other carotenoids **(A)**, and grain yield and other agronomic traits **(B)** of 60 provitamin A inbred lines used in line ×tester crosses evaluated across eight environments in Nigeria in 2020 and 2021.

In the combined analysis of variance, environment and hybrid had significant effects on all carotenoids (Table 1). There were significant line ×environment, tester ×environment, and line ×tester ×environment interaction mean squares for most of the carotenoids in our study. The GCA effects among inbred lines and between testers as well as the SCA effects were significant for all carotenoids (Table 1). The proportional contribution of line, tester, and line ×tester to the total genotypic variance for all carotenoids varied from 2 to 16%, 80–98%, and 0.4–40%, respectively. The repeatability values varied from 0.65 to 0.95 for all carotenoids (Table 1). Baker’s ratios for carotenoids varied from 0.98 to 0.99 (Table 1). As shown in Figure 1A, the contributions of the additive gene effects were greater than 80% for most of the carotenoids.

**TABLE 1:** Mean squares from the combined analysis of variance of provitamin A and other carotenoids of testcrosses of 60 provitamin A–enriched maize inbred lines and two testers evaluated across four environments in Nigeria in 2020 and 2021.

Source of variation	DF	Lutein	Zeaxanthin	β-Cryptoxanthin	α-Carotene	β-Carotene	Provitamin A
Env	3	722.67**	385.65**	12.51**	2.57**	829.52**	909.02**
REP (Env)	4	77.73**	93.37**	9.3**	1.05**	34.71**	57.67**
Block (Env × Rep)	240	4.64**	5.88**	0.4**	0.08**	2.48**	3.04**
Hybrid (H)	123	28.6**	61.85**	8.35**	0.18**	34.27**	25.83**
Testcross	119	29.25**	60.92**	11.31**	2.25**	34.16**	25.14**
Line (GCA)	59	75.13**	85.35**	15.26**	0.34**	50.32**	45.35**
Tester (GCA)	1	367.23**	5215.77**	388.35**	2.58**	2438.68**	1498.54**
Line × tester (SCA)	59	15.11**	10.11**	1.63**	0.1**	6.22**	5.94**
Hybrid × Env	369	3.45**	3.31	0.37*	0.06**	3.15**	3.25**
Line × Env	177	4.41*	5.22	0.53	0.09**	4.97**	5.17**
Tester × Env	3	66.89**	42.52**	7.39**	0.73**	67.63**	85.09**
Line × tester × Env	177	4.71*	4.54	0.47*	0.07	3.07**	3.48**
Error	252	2.11	764.68	0.29	0.04	1.29	1.36
Repeatability		0.89	0.94	0.95	0.65	0.91	0.87
CV (%)		18.41	17.94	13.59	24.42	12.63	10.25
Baker ratio		0.98	0.99	0.99	0.98	0.99	0.99

DF, degree of freedom *, ** significant at probability <0.05 and 0.01 levels, respectively.

The combined analysis of variance also revealed significant environmental effects on grain yield and other agronomic traits (Table 2). The differences among hybrids and hybrid ×environment interactions were significant for grain yield and other agronomic traits (Table 2). Significant GCA effects were found among the PVA inbred lines and between the two testers for grain yield and other desirable agronomic traits (Table 2). The variations in SCA effects were also significant for grain yield and other agronomic traits. The line × environment interaction mean squares were not significant for grain yield and most measured agronomic traits, whereas the tester ×environment interaction mean squares were significant for grain yield and most of the major agronomic traits. The line ×tester ×environment interactions were not significant for all measured agronomic traits (Table 2). The proportional contribution of line, tester, and line ×tester to the total genotypic variance for grain yield and other agronomic traits varied from 5 to 35%, 44–93%, and 2–23%, respectively. Repeatability estimates for grain yield and other agronomic traits varied from 0.72 to 0.92 (Table 2). The relative importance of the GCA mean squares over the SCA mean squares varied from 0.87 to 0.99 for grain yield and other agronomic traits (Table 2). Also, the contribution of the additive gene effects was greater than that of the non-additive gene effects for grain yield and all measured agronomic traits (Figure 1B).

**TABLE 2:** Mean squares from the combined analyses of variance of grain yield and other agronomic traits of testcrosses of 60 provitamin A–enriched maize inbred lines and two testers evaluated across eight environments in Nigeria in 2020 and 2021.

Source of variation	DF	Grain yield	Days to tasseling	Days to silking	Plant height	Ear height	Plant aspect	Ear aspect
Env	7	711565530**	4052.42**	3470.24**	95126.36**	8507.51**	5.58**	15.28**
REP (Env)	8	17729670**	13.01**	14.32**	888.45**	626.08**	0.34	0.82**
Block (Env × Rep)	480	1112531	2.09**	2.4**	163.36**	123.04**	0.22	0.16
Hybrid (H)	123	7278902**	15.2**	16.11**	1298.19**	748.63**	0.75**	1**
Testcross	119	7221635**	14.72**	15.50**	1226.83**	731.19**	0.77**	0.97**
Line (GCA)	59	10025419**	29.98**	32.14**	2948.56**	1664.49**	1.14**	1.29**
Tester (GCA)	1	21472764**	504.27**	470.06**	17708.19**	10343.3**	1.46*	3.58**
Line × tester (SCA)	59	9365602**	8.4**	9.69**	430.96**	247.19**	0.69**	1.16**
Hybrid × Env	860	1592711**	1.75**	1.9*	146.29**	94.81**	0.23*	0.19*
Line × Env	177	1961929	2.35	2.52	211.3**	137.75**	0.27	0.23
Tester × Env	3	19595613**	6.32	7.49	4137.21**	673.71**	1.17**	0.35
Line × tester × Env	177	1314650	1.63	1.86	176.34	85.6	0.27	0.16
Error	503	1136506	1.43	1.57	97.92	70.25	0.18	0.16
Repeatability		0.82	0.92	0.92	0.91	0.91	0.72	0.84
CV (%)		17.19	2.1	2.14	5.16	9	17.09	15.76
Baker ratio		0.87	0.99	0.99	0.99	0.99	0.88	0.89

DF, degree of freedom *, ** significant at probability <0.05 and 0.01 levels, respectively.

The tester with low provitamin A content (T2) displayed slightly higher genetic variance estimates for β-carotene and provitamin A content (Table 3). On the other hand, the two testers (T1 and T2) showed similar genetic variances for lutein, zeaxanthin, β-cryptoxanthin, and α-carotene. The genetic variances for the tester with high provitamin A content (T1) were the highest for grain yield, days to anthesis, days to silking, and ear aspect, whereas those for T2 were highest for plant height, ear height, and plant aspect scores (Table 3).

**TABLE 3:** Genetic variance and standard error between testcrosses obtained for the testcrosses of each tester (T1 and T2) evaluated at four locations in 2020 and 2021.

Traits	Genetic variance ± standard error	
	T1	T2
Grain yield (kg/ha)	7.776 ± 313.802	4.031 ± 253.711
Days to anthesis	10.021 ± 0.413	7.81 ± 0.373
Days to silking	10.624 ± 0.432	7.997 ± 0.389
Plant height (cm)	9.379 ± 3.478	10.121 ± 3.941
Ear height (cm)	7.905 ± 2.874	9.415 ± 2.717
Plant aspect (1–5)	3.093 ± 0.077	4.164 ± 0.096
Ear aspect (1–5)	7.312 ± 0.111	4.495 ± 0.09
Lutein (µg/g)	9.613 ± 0.671	10.582 ± 0.547
Zeaxanthin (µg/g)	9.708 ± 0.564	9.811 ± 0.681
β-Cryptoxanthin (µg/g)	16.966 ± 0.252	16.552 ± 0.274
α-Carotene (µg/g)	2.473 ± 0.036	2.694 ± 0.049
β-Carotene (µg/g)	6.578 ± 0.567	8.293 ± 0.377
Provitamin A (µg/g)	5.253 ± 0.51	7.496 ± 0.4

### Carotenoid content and agronomic performance of testers and their testcrosses

Provitamin A and other carotenoid content as well as agronomic performance of the 120 testcrosses are presented in Supplementary Table S2. The testcrosses accumulated between 6.2 and 18.4 μg/g of provitamin A in their grains. Amongst these, 58 testcrosses of T1 and 45 testcrosses of T2 accumulated as much PVA as or significantly higher PVA than the best commercial provitamin A–biofortified hybrid, that is, Ife Hybrid-4. Moreover, 44 testcrosses of T1 and 10 testcrosses of T2 had 24–95% higher PVA concentrations than the cross between the two testers (T1 × T2). None of the 60 testcrosses of T1 and 59 testcrosses of T2 accumulated significantly less PVA than T1 × T2 (Supplementary Table S2). Over 90% of the testcrosses involving the two testers had mean lutein, zeaxanthin, β-cryptoxanthin, α-carotene, and β-carotene content that did not differ significantly from or significantly higher than that of T1 × T2.

Testcrosses of T1 had mean grain yields varying from 3,994 to 7,906 kg/ha, whereas those of T2 had grain yields ranging from 4,206 to 7,618 kg/ha (Table 4, Supplementary Table S2). A total of 32 testcrosses of T1 and 38 testcrosses of T2 did not differ significantly from the best hybrid check, Ife Hybrid-4, in their mean grain yields. A total of 50 testcrosses of T1 and 57 testcrosses of T2 produced as high as or 16–30% higher grain yields than the cross between the two testers (T1 × T2). Amongst the testcrosses showing competitive or better grain yields than Ife Hybrid-4, 10 testcrosses involving T1 and one testcross involving T2 accumulated 13.0–15.1 μg/g of provitamin A. The observed increases in the provitamin A content in these hybrids over Ife Hybrid-4 varied from 14 to 33%. The tester T1 had higher minimum, maximum, and mean lutein, β-carotene, and PVA concentrations compared to T2 (Table 4). In addition, the high PVA tester had a broader range for grain yield (Table 4). Almost all the testcrosses had anthesis and silking dates that were similar to or 4 days later than that of T1 × T2 (Supplementary Table S2). More than 75% of the testcrosses had the same as or significantly higher plant height and ear placement than T1 × T2. Also, more than 95% of the testcrosses had desirable plant and ear aspect scores (≤3.0).

**TABLE 4:** Minimum, maximum, and mean values of agronomic traits and carotenoids for the testcrosses of two testers evaluated across eight environments.

Trait	T1			T2		
	Min	Max	Mean	Min	Max	Mean
Grain yield (kg/ha)	3994.73	7906.10	6087.90	4206.17	7618.21	6293.15
Days to anthesis	55.13	59.81	57.49	54.19	58.47	56.45
Days to silking	56.38	61.31	59.05	55.63	60.00	58.05
Plant height (cm)	177.69	213.81	194.32	165.31	213.44	188.29
Ear height (cm)	80.31	113.88	95.26	77.81	107.25	90.63
Plant aspect (1–5)	1.88	3.17	2.50	1.96	3.25	2.57
Ear aspect (1–5)	1.81	3.34	2.59	1.91	3.00	2.50
Lutein (µg/g)	4.32	19.46	8.53	4.31	14.92	7.31
Zeaxanthin (µg/g)	1.78	12.70	7.30	6.05	17.71	11.95
β-Cryptoxanthin (µg/g)	1.36	5.84	3.32	2.45	6.90	4.59
α-Carotene (µg/g)	0.51	1.23	0.79	0.53	1.48	0.89
β-Carotene (µg/g)	6.41	16.84	10.69	4.16	12.40	7.49
Provitamin A (µg/g)	8.43	18.42	12.74	6.20	15.07	10.23

As shown in Supplementary Table S3, 33 testcrosses of T1 and 38 testcrosses of T2 displayed positive standard heterosis of 1–30% for grain yield (Supplementary Table S3). Amongst these, 15 testcrosses of T1 and 24 testcrosses of T2 had standard grain yield heterosis of 10–30%. For provitamin A content, 58 testcrosses involving T1 showed standard heterosis varying from 9 to 96%, while 42 testcrosses involving T2 displayed standard heterosis varying from 1 to 60%. It is noteworthy to highlight that amongst the testcrosses showing at least 10% standard heterosis for grain yield, all 15 testcrosses of T1 displayed standard heterosis of 22–60% for provitamin A content while only 11 testcrosses of T2 showed standard heterosis of 10–60%. The two provitamin A–enriched commercial hybrids (Ife Hybrid-3 and Ife Hybrid-4) had standard heterosis of 6–16% for grain yield and 20–21% for provitamin A content. The number of testcrosses involving T1 showing positive standard heterosis was 43 for lutein, 43 for zeaxanthin, and 58 for β-carotene, whereas those involving T2 with positive standard heterosis were 29 for lutein, 60 for zeaxanthin, and 31 for β-carotene (Supplementary Table S3).

Correlation analysis showed significant but small negative genotypic and phenotypic correlations between grain yield and lutein, β-carotene, and provitamin A content (Supplementary Table S4). In contrast, the genotypic and phenotypic correlations between grain yield and zeaxanthin were significant and positive. Zeaxanthin was positively correlated with β-cryptoxanthin and α-carotene but negatively correlated with provitamin A and β-carotene content (Supplementary Table S4).

### Combining ability estimates for provitamin A–enriched inbred lines and testers

The high provitamin A tester (T1) had significant and positive GCA effects for lutein, β-carotene, and provitamin A but had significant and negative GCA effects for zeaxanthin and β-cryptoxanthin (Table 5). In contrast, the low provitamin A tester (T2) had significant and positive GCA effects for zeaxanthin and β-cryptoxanthin but had significant and negative GCA effects for lutein, β-carotene, and provitamin A content. T1 had negative but not significant GCA effects for grain yield but had significant and positive GCA effects for days to anthesis, days to silking, and plant and ear height. T2 had positive but not significant GCA effects for grain yield but had significant negative GCA effects for days to anthesis, days to silking, and ear height (Table 5).

**TABLE 5:** Estimates of general combining ability (GCA) effects for two testers evaluated across eight environments.

Traits	T1	T2
GY (kg/ha)	−107.71	108.28
DYANTH (days)	0.51**	−0.52**
DYSK (days)	0.5**	−0.5**
PHT (cm)	2.99	−3
EHT (cm)	2.3*	−2.3*
PASP (1–5)	−0.03	0.03
EASP (1–5)	0.04	−0.04
Lutein (µg/g)	0.62*	−0.62*
Zeaxanthin (µg/g)	−2.33**	2.33**
β-Cryptoxanthin (µg/g)	−0.64**	0.64**
α-Carotene (µg/g)	−0.05	0.05
β-Carotene (µg/g)	1.59**	−1.59**
Provitamin A (µg/g)	1.25**	−1.25**

*, ** significant at probability <0.05 and 0.01 levels, respectively.

Estimates of GCA effects of the provitamin A–enriched maize inbred lines for grain yield, other agronomic traits, and carotenoids are presented in Supplementary Table S5. A total of 26 inbred lines had positive GCA effects for grain yield, whereas 27 lines had positive GCA effects for provitamin A content (Supplementary Table S5). Amongst these, 10 lines (TZI2012, TZI2142, ATZI2130, TZI2065-2, TZI2161, TZI2025, TZI1278, TZI1314, TZI1304, and TZI2032) showed a positive GCA effect for both grain yield, and provitamin A content. It is worth mentioning that most of the lines with significant positive GCA effects for provitamin A content had negative GCA effects for grain yield (Supplementary Table S5).

Amongst all testcrosses, 27 inbred lines crossed to T1 and 23 inbred lines crossed to T2 had positive SCA effects varying from 0.10 to 1.67 μg/g of provitamin A (Supplementary Table S6). In addition, 24 inbred lines each crossed to T1 and T2 exhibited moderate (SCA effects ≥ 100 kg/ha) to significant positive SCA effects for grain yield (Supplementary Table S6). Out of the inbred lines with positive SCA effects for grain yield, eight inbred lines crossed to T1 and seven inbred lines crossed to T2 also had positive SCA effects for the PVA content. Only three inbred lines, namely, TZI2025, TZI2024, and TZI2156 in crosses with T1 combined significant and positive SCA effects for grain yield with positive SCA effects for the provitamin A content (Supplementary Table S6).

## Discussion

Progress in the development and deployment of new maize hybrids combining high yields with enhanced concentrations of provitamin A hinges on the identification and use of desirable testers. A good tester in maize breeding should be simple to use, provide information that correctly classifies the performance of lines under evaluation, and maximizes genetic gain (Hallauer et al., 1988). The current study was thus conducted to assess the usefulness of inbred two testers with contrasting provitamin A content in revealing genetic differences among provitamin A–enriched maize inbred lines. A total of 60 maize inbred lines with varying concentrations of provitamin A were evaluated in crosses with the two testers in four test environments for carotenoid content and eight test environments for agronomic performance in Nigeria. The results showed that both carotenoid content and agronomic performance of the testcrosses were significantly affected by the differences in environmental factors arising from prevalent differences in the amount and distribution of rainfall, temperature, and nutrient content of the soil as well as moisture-holding capacity of soil during testcross evaluation. Although the line ×tester ×environment interactions were significant for two of the five carotenoids and provitamin A, their interaction mean squares were 3–18 times smaller than the corresponding mean squares for testcrosses. Also, none of the agronomic traits displayed significant line ×tester × environment interactions. These results demonstrate the dominant role of the genetic backgrounds of the testcrosses in defining their agronomic performance and carotenoid content across test environments, which is reflected in the observed high repeatability values for all carotenoids and agronomic traits.

The significant GCA and SCA effects and high baker’s ratio values for carotenoid concentrations, grain yield, and other agronomic traits suggest that the expressions of these traits are controlled by both additive and non-additive gene effects. The contribution of the additive gene effects was, however, greater than the contribution of the non-additive gene effects to the observed total genetic variance for both carotenoid content and agronomic traits. The preponderance of additive gene effects highlights the feasibility of early generation evaluation and selection of high-yielding inbreds with high provitamin A content that can subsequently be used as parents of superior hybrids. The prominence of additive gene effects in the inheritance of carotenoid concentrations and agronomic traits also indicates that populations formed from the most promising provitamin A–enriched maize inbred lines identified in the present study could be invaluable sources of new inbred lines with desirable agronomic features and much higher levels of provitamin A and other carotenoids. The best-inbred lines can also be crossed with other inbred lines possessing desirable agronomic traits and different carotenoid profiles to develop a new generation of lines combining superior agronomic traits and high concentrations of provitamin A and other beneficial carotenoids.

The choice of a suitable tester in hybrid breeding programs is determined by the capacity of the tester to discriminate among new maize inbred lines under evaluation. The observed significant line × tester interaction for carotenoids and agronomic traits in the present study indicates that the two testers were effective in discriminating the provitamin A–enriched maize inbred lines across environments. These results are consistent with the findings of other studies assessing the combining ability, carotenoid content, and agronomic performance of maize inbreds in hybrid combinations (Menkir et al., 2014; Owens et al., 2014; Suwarno et al., 2014; Annor and Badu-Apraku, 2016; Owusu et al., 2017). Additional critical factors for identifying suitable testers include GCA effects, magnitude of genetic variance, frequency of favorable alleles for the target traits, and average testcross performance (Sprague and Tatum, 1942; Guimarães et al., 2012). In the current study, T1 had significant and positive GCA effects for β-carotene and PVA content and thus has favorable alleles for these traits with additive effects. Moreover, the testcrosses involving T1 had more PVA content compared with the testcrosses involving T2, suggesting that the choice of a tester with high provitamin A content can impact higher provitamin A content in its hybrids.

The low provitamin A tester (T2) had slightly higher genetic variance for β-carotene and provitamin A content, suggesting that its unfavorable alleles allowed better expressions of favorable alleles controlling provitamin A levels in the 60 maize inbred lines evaluated. Other studies have also reported that testers with a low frequency of favorable alleles would be effective in eliciting genetic differences among inbred lines (Rawlings and Thompson, 1962; Hallauer and Lopez-Perez, 1979). Nonetheless, significant and large genetic variances were also found among testcrosses involving the high provitamin A tester (T1) for β-carotene and provitamin A content, indicating its effectiveness in discriminating the provitamin A–enriched maize inbred lines evaluated in our study. The two testers also showed comparable genetic variances for other carotenoids, indicating that T1 and T2 were effective in characterizing the other carotenoid content in testcrosses. The results of analyses of genetic variances and GCA effects suggest that T1 can be considered a suitable tester for identifying promising provitamin A–enriched parental lines to develop superior provitamin A–biofortified hybrids for deployment. The low PVA tester that displayed positive GCA effects for grain yield, zeaxanthin, and β-cryptoxanthin and negative GCA effects for plant height and ear heights can also be used as a second potential tester to evaluate maize inbred lines for agronomic performance and non-provitamin A carotenoid content.

Estimating the GCA effects of inbred lines is also important for selecting potential parents to develop high-yielding hybrids and synthetic varieties with high provitamin A content. Eight inbreds with significant positive GCA effects for provitamin A content displayed negative GCA effects for grain yield possibly due to the negative correlation (r = −0.25, *p* < 0.01) between these traits arising from the dilution effects of different yield potentials of the testcrosses. Nevertheless, 10 inbred lines had positive GCA effects for both provitamin A content and grain yield in the present study, suggesting that these inbreds possess favorable alleles for use to improve agronomic performance and provitamin A content in new inbred lines derived from source populations. Furthermore, the 16 inbred lines with positive GCA effects (four inbreds with significant positive GCA effects and 14 inbreds with non-significant positive GCA effects) for grain yield but negative GCA effects (five inbreds with significant negative GCA effects and 13 inbreds with non-significant positive GCA effects) for provitamin A content can be exploited as parental lines to increase the frequency of favorable alleles for grain yield in tropical maize breeding programs.

The significant SCA effects for provitamin A content recorded for two of each of T1 and T2 testcrosses implies that crossing specific pairs of parental lines can optimize provitamin A concentrations in their hybrids. The two testcrosses involving T2 also had moderate (159 and 173 kg/ha) and positive SCA effects for grain yield and can thus be used as potential female parents for developing three-way cross hybrids with high yield potential and a higher levels of PVA. Many testcrosses included in the present study combined grain yields and provitamin A content comparable to or surpassing the best commercial hybrid marketed in Nigeria (Ife Hybrid-4). All of the top 10 highest yielding testcrosses out-yielded Ife Hybrid-4. Four of the top 10 highest yielding testcrosses had PVA concentration surpassing the PVA level of Ife Hybrid-4. Two testcrosses (TZI 2025 × T1 and TZI1715 × T2) with grain yields of 7,256 and 7,257 kg/ha, respectively, had 15.0 μg/g provitamin A content that met the target set by the Harvestplus Challenge Program (https://www.harvestplus.org/crop-development/, accessed on 29 June 2022).

The observed high level of standard heterosis for grain yield and provitamin A content in 25 testcrosses of the provitamin A–enriched maize inbred lines and the two testers indicated that the inbred parents carry favorable complementary alleles to the two testers. Also, many testcrosses exhibited high positive heterosis for lutein, zeaxanthin, and β-carotene content. These results further demonstrate the possibility of simultaneously improving grain yield and provitamin A content in maize hybrids. The weak (the coefficients of determination (R^2^) = 0.06) but significant negative correlation (r = −0.25, *p* < 0.01) between grain yield and provitamin A content confirms the feasibility of developing high-yielding hybrids with considerably high levels of provitamin A content. These findings were different from those of Menkir et al. (2014) and Halilu et al. (2016), who reported a positive but non-significant correlation between grain yield and PVA content. Further studies involving inbred lines with diverse genetic backgrounds may be necessary to elucidate the type of relationship between the two traits.

## Conclusion

Our study found the two inbred testers were successful in discriminating the provitamin A–enriched maize inbred lines. There was a significant genetic variation among the hybrids for all carotenoids, grain yield, and other agronomic traits. The high provitamin A tester that exhibited positive GCA effects for β-carotene and PVA concentrations, broader testcross performance in grain yield and other agronomic traits, and higher levels of provitamin A in testcrosses can be considered as an appropriate tester for breeding programs targeting the development of superior provitamin A–biofortified hybrids. The tester can also be used in separating provitamin A–enriched maize inbred lines into heterotic groups to maximize the expression of heterosis in hybrids. The 10 best inbred lines (TZI2012, TZI2142, ATZI2130, TZI2065-2, TZI2161, TZI2025, TZI1278, TZI1314, TZI1304, and TZI2032) with positive GCA effects for grain yield and provitamin A content identified in the present study could be used as parental lines to form high-yielding single cross hybrids and synthetic varieties with high concentrations of provitamin A. Also, the best testcrosses with good SCA effects for provitamin A content and grain yield could be used as parents to develop three-way-cross hybrids with superior agronomic performance and enhanced concentration of provitamin A.

## Data Availability

The original contributions presented in the study are included in the article/Supplementary Material; further inquiries can be directed to the corresponding author.
